# Inhibitory Effects of Pectic Polysaccharide Isolated from *Diospyros kaki* Leaves on Tumor Cell Angiogenesis via VEGF and MMP-9 Regulation

**DOI:** 10.3390/polym13010064

**Published:** 2020-12-26

**Authors:** Jun Yeon Park, Myoung-Sook Shin

**Affiliations:** 1 Department of Food Science and Biotechnology, Kyonggi University, Suwon-si, Gyeonggi-do 16227, Korea; rhemf@kgu.ac.kr; 2 College of Korean Medicine, Gachon University, Seongnam-si, Gyeonggi-do 13120, Korea

**Keywords:** angiogenesis, pectic polysaccharide, MMP-9, VEGF

## Abstract

Persimmon leaves are an attractive source of phytochemicals with potential health benefits. However, there are only a few reports on the chemical properties and biological activity of the polysaccharide fractions (PLE-I–III) of persimmon leaves. We evaluated the angiogenesis-inhibiting ability of pectic-polysaccharides. The molecular weight of PLEs was determined using a high-performance size-exclusion chromatography system. Tube formation assay of human umbilical vein endothelial cells (HUVECs) was performed using Matrigel-coated 96-well plates. Matrix metalloproteinase (MMP-9), vascular endothelial growth factor (VEGF), PI3K, Akt, and p38 phosphorylation levels were determined using Western blotting; VEGF and MMP-9 transcript levels were measured using SYBR Green qRT-PCR. PLE-I–III significantly inhibited HUVEC tube formation at 12.5 and 25 μg/mL. Among them, PLE-II showed the strongest anti-tube formation activity, and the mRNA/protein expression of angiogenesis-related factors (VEGF/MMP-9) was significantly reduced by PLE-II. PLE-II also suppressed the phosphorylation of PI3K/AKT and p38, JNK, and NF-κB p65 in HUVECs. These results suggest that the polysaccharide PLE-II isolated from persimmon leaves inhibited VEGF and MMP-9 expression in HUVECs via regulation of PI3K/AKT, p38, JNK, and NF-κB p65 signaling pathways.

## 1. Introduction

Angiogenesis, which is the process of the formation of new capillaries, is essential for the proliferation of primary or metastatic tumors and plays an important role in the physiological processes of normal tissue, such as growth, reproduction, and wound healing [1]. As solid tumors require oxygen and nutrients for growth, they are angiogenesis-dependent and cannot grow without local angiogenesis [2]. Angiogenesis requires various processes such as endothelial cell growth as well as basement membrane invasion, migration, and differentiation of endothelial cells [2,3]. From this point of view, a mechanism that inhibits the growth of vascular endothelial cells or processes such as infiltration and migration would be effective in blocking angiogenesis and preventing tumor growth. Angiogenesis is regulated by various growth factors such as basic fibroblast growth factor (bFGF, vascular endothelial growth factor (VEGF), and angiogenin [4]. Of these growth factors, VEGF is a potent direct modulator of angiogenesis and is involved in inflammation, fibrosis, wound healing, and cancer [5]. In the formation of secondary tumors via the dissemination of metastatic cancer cells moving through blood vessels or lymphatic vessels, degradation of the extracellular matrix and basement membrane occurs. Matrix metalloproteinases (MMPs) are classified as type IV collagenases (MMP-2 and MMP-9), stromelysin (MMP-3), and interstitial collagenase (MMP-1) according to their substrate preference [6,7,8]. MMP-2 and MMP-9 play essential roles in metastasis and the expression of these enzymes is most closely related to tumor metastasis and invasion ability [9,10].

Pectic polysaccharides that exist in the middle lamellar and primary cell walls of plants, are a group of polymers that contain 1,4-linked α-D-galacturonic acid (GalA) residues, called homogalacturonan, glycosidically interlinked with rhamnogalacturonan-I (RG-I) and rhamnogalacturonan-II (RG-II) [11,12,13]. Pectic polysaccharides have previously been used only as gel agents and stabilizers and considered useless because of their indigestible properties. However, some studies have examined their structure and pharmacological activities [11]. RG-I and RG-II isolated from natural products have been reported to possess pharmacological activities such as complement activating activity, stimulation of macrophages [14], bone marrow cell proliferation mediated by intestinal immune system [15], natural killer (NK) cell cytotoxicity, including cytolytic activity against cancer cell lines [16,17], and anti-metastatic effects in vivo [18,19].

The leaves of persimmon (*Diospyros kaki* Thumb.) are widely accepted as edible, and their pharmacological effects such as anti-allergic and anti-tumor have been attributed to tannins, flavonoids, and organic acids [20,21]. Therefore, in this study, we aim to investigate the effect of pectic polysaccharides on angiogenesis, an early process of cancer metastasis. Therefore, we investigated the effects of polysaccharides (PLE-I–III) isolated from *Diospyros kaki* (persimmon) leaves on tube formation and identified related proteins as well as signal pathways using HUVECs.

## 2. Materials and Methods

### 2.1. Antibodies and Reagents

Antibodies against VEGF (A-20), MMP-9 (H-129), and β-actin (I-19) were purchased from Santa Cruz Biotechnology (Santa Cruz, CA, USA). In addition, antibodies against PI3K Class III (D4E2), phospho-PI3K Class III (Ser 249), AKT (5G3), phospho-AKT (Ser473), p38 (D13E1), phospho-p38 Thr180/Tyr182 (D3F9), ERK (p44/42), phospho-ERK (Thr202/Tyr204), phospho-p65 (Ser536) (93H1), p65 (C22B4), phospho-JNK (Thr183/Tyr185), JNK, and GAPDH were purchased from Cell Signaling Technology (Danvers, MA, USA). EGM^TM^-2 Endothelial Cell Growth Medium-2 BulletKit^TM^ was purchased from Lonza (Walkersville, MD, USA). The EZ-Cytox cell viability assay kit and EZ-LDH cytotoxicity assay kit were purchased from DoGenbio (Seoul, Korea). Recombinant human tumor necrosis factor (TNF)-α was purchased from Peprotech (Rocky Hill, NJ, USA). Mayer’s hematoxylin solution was purchased from Muto Pure Chemicals (Tokyo, Japan).

### 2.2. Preparation of Polysaccharide Fractions (PLE-I-III) from Persimmon Leaves

**Figure 1:**
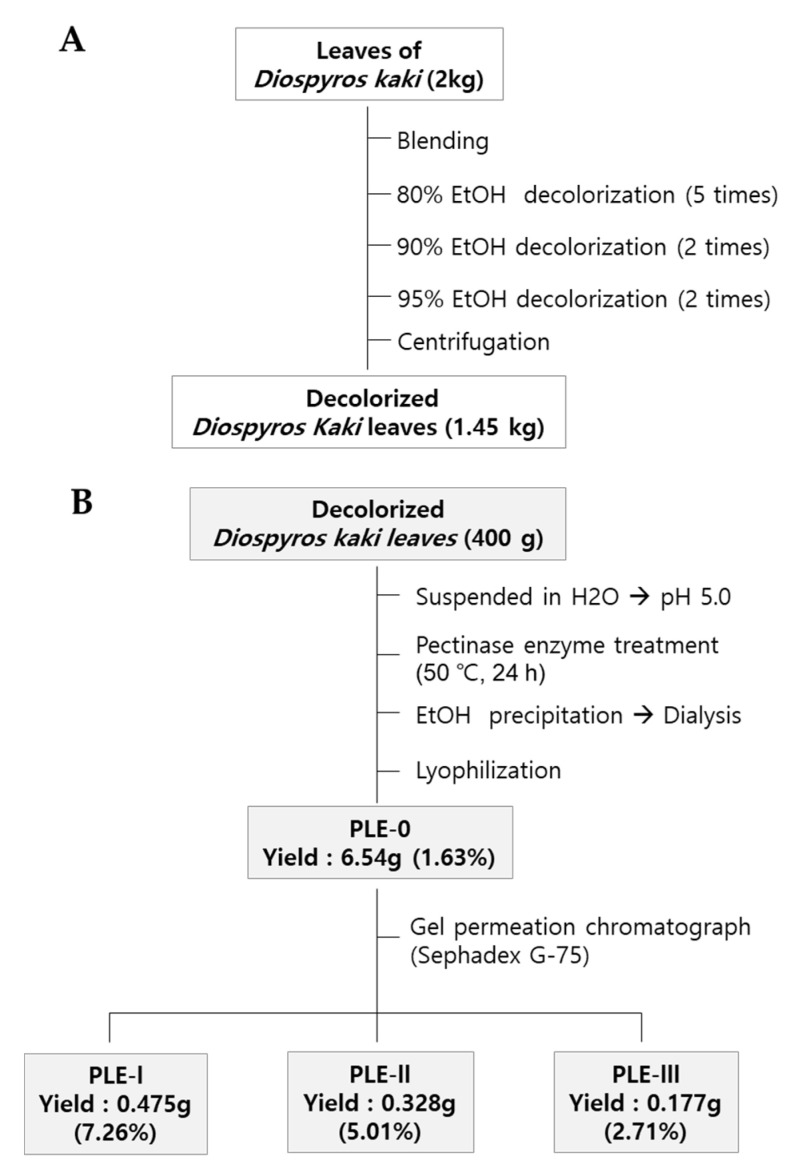
Isolation and fractionation procedure for PLE-0 polysaccharide (**A**), -I, -II, and –III polysaccharide (**B**) from persimmon leaves.

Dried persimmon leaves (2 kg) were harvested in Geochang-gun, Gyeongnam-do, Korea, and pulverized with a mechanical grinder. In brief, the pulverized leaves were soaked with 80% EtOH, and the supernatant was removed for decolorization. The decolorization process was repeated with an increased concentration of EtOH (80–95%). After centrifugation, the precipitate was collected and dried using a convection oven (1.45 kg) (Figure 1A). To obtain crude polysaccharides, dried precipitant (400 g) was suspended in distilled water (4 L) and hydrolyzed using pectinase (50 °C, pH 5.0) for 24 h. After the inactivation of the enzyme by boiling, the mixture was centrifuged at 6000 *× g* for 30 min to collect the supernatant. Then, four volumes of EtOH were added to the supernatant, and the resulting precipitate was collected by centrifugation. Finally, the precipitate was dissolved in distilled water and dialyzed to remove EtOH or low-molecular-weight materials (molecular cut off: 14,000 Da) and then lyophilized (PLE-0). PLE-0 was fractionated by gel permeation column chromatography using Sephadex G-75 (GE Healthcare Life Sciences). The procedures for obtaining three polysaccharides (PLE-I, -II, and -III) and the yield of each fraction are described in Figure 1B. The elution profiles of PLE-0 determined using gel permeation column chromatography and the molecular weights of PLE-0, -I, -II, and -III are shown in Supplementary Figure S1. More detailed information on the molecular weight of each fraction has been described in our previous publication [22].

### 2.3. Cell Culture

HUVECs were purchased from ATCC (Manassas, VA, USA) and maintained using EGM^TM^-2 Endothelial Cell Growth Medium-2 BulletKit^TM^ containing various growth factors such as human FGF, VEGF, insulin-like growth factor, EGF, hydrocortisone, ascorbic acid, 5% FBS, and penicillin/streptomycin at 37 °C in a humidified atmosphere (5% CO_2_, 95% air). The cells were sub-cultured every 3 days. The cells were sub-cultured every 3 days to maintain monolayer cells.

### 2.4. Viability Measurements of HUVECs

The cytotoxicity of PLE-I, -II, and -III polysaccharides in HUVECs was measured using the MTT-based EZ-Cytox reagent or lactate dehydrogenase (LDH) cytotoxicity assay kit (EZ-LDH). HUVECs were seeded at a density of 2.0 × 10^4^ cells/well in a 96-well plate. The cells were then treated with various concentrations of PLE-I–III or phosphate-buffered saline (PBS) to serve as the control and incubated at 37 °C in a humidified atmosphere. After 24 h incubation, HUVEC viability was estimated using a FilterMax F5 microplate reader (Molecular Devices, San Jose, CA, USA).

### 2.5. Tube Formation Assay

The 96-well culture plates were coated with Matrigel (10 mg/mL, 60 μL/well) for 1 h at 37 °C. Then, 50 µL of PLE-I, -II, and -III solution mixed with HUVECs (1.5 × 10^4^ cells) was added to the Matrigel-coated 96-well plate and incubated for 24 h. After incubation, the plate was washed once with distilled water. Then, the cells were fixed with 4% paraformaldehyde for 30 min, followed by staining with Mayer’s hematoxylin for 20 min at room temperature. Thereafter, the cells were washed with distilled water and 70% EtOH (2 times) and finally dried for observation under a microscope. Cell morphology and tubular structure formation were observed using a light microscope. The degree of tube formation was quantified by measuring the lengths of the tubes in the captured images using the ImageJ program.

### 2.6. Real-Time Quantitative Reverse Transcription PCR

Total RNA from HUVECs or Colon 26 M3.1 cells was isolated and purified using the RNeasy Mini kit (Qiagen, Valencia, CA, USA), and cDNA was prepared using RevertAid First Strand cDNA Synthesis kit (Fermentas, MA, USA) according to the manufacturer’s protocol. To amplify the cDNA, reverse transcription of cDNA was performed by real-time quantitative reverse transcription PCR (qRT-PCR) using SYBR Green PCR Master Mix (Applied Biosystems, Foster City, CA, USA) with the indicated primers (Table 1). Data were analyzed according to the comparative *Ct* method and were normalized to human β-actin against human VEGF or MMP-9. qRT-PCR was performed using the Real-Time PCR 7500 system (Applied Biosystems, USA).

### 2.7. Preparation of Cell Lysates for Immunoblotting

HUVECs were treated with PLE-II at the indicated concentrations for 24 h. After treatment, the cells were washed with PBS and lysed in cold RIPA buffer (Rockland, Limerick, PA, USA) supplemented with 1 mM DTT (Merck, Darmstadt, Germany) and diluted protease inhibitor cocktail tablets (Sigma, St. Louis, MO, USA). After centrifugation, the amount of protein in each supernatant was quantified, mixed with SDS-sample buffer, and denatured for 5 min at 95 °C. Electrophoresis was performed using 10–12% tris-glycine SDS-polyacrylamide gel. Protein bands were transferred to polyvinylidene fluoride membranes, which were blocked at room temperature with 5% skim milk. After three washes with tris-buffered saline containing 0.1% Tween^®^ 20, the membranes were incubated with a specific antibody for 3 h, followed by washes and incubation with a secondary antibody. The protein bands were visualized with the SuperSignal^TM^ West Pico PLUS Chemiluminescent substrate (Thermo Scientific, Rockford, IL, USA) using the Fusion Solo System (Vilber Lourmat, Paris, France).

### 2.8. Gelatin Zymography

Pre-cast zymogram gel (10% SDS-PAGE gel containing 0.1% gelatin) and buffer kits were purchased from KOMA BIOTECH (Seoul, Korea). HUVECs were cultured in 6-well plates with complete media, and then, the cells were synchronized with FBS-free media for 6 h. HUVECs were treated with the indicated concentrations of PLE-II for 24 h, and the medium was collected for zymogram electrophoresis. The protein concentrations of the medium (sample) were adjusted to equal levels using the BCA assay. A sample was mixed with a 2× sample buffer and separated using a pre-cast zymogram gel by electrophoresis. The gel was washed with zymogram renaturing and developing buffers according to the manufacturer’s protocol. To analyze MMP-9 activity, the gels were stained with 0.5% Coomassie Blue R-250 solution and destained with destaining buffer (40% methanol and 10% acetic acid). Images were obtained using the Fusion Solo System (Vilber Lourmat).

### 2.9. Statistical Analysis

The results are expressed as the mean ± standard deviation (SD) of triplicate experiments. Statistical significance was determined using the non-parametric Mann–Whitney U (unpaired) test, with *p* < 0.01 or *p* < 0.05 considered significant. All statistical tests were performed using the statistical package for Prism 8 (GraphPad Software, version 9, San Diego, CA, USA).

## 3. Results and Discussion

### 3.1. Chemical Properties of Polysaccharide Fractions (PLE-0, I–III) Isolated from Persimmon Leaves

**Figure 2:**
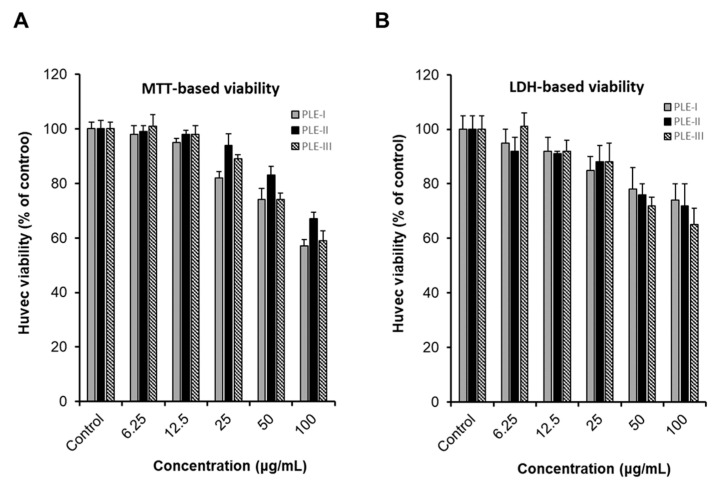
The cytotoxic effect of PLE-I–III on human umbilical vein endothelial cells (HUVECs). Comparison of the effect of PLE-I–III polysaccharide fractions on HUVEC proliferation and assessment of cytotoxicity. The cells were treated with the indicated concentrations of PLE-I–III for 24 h, and then, cell viability was evaluated using the EZ-Cytox reagent (**A**) or LDH reagent (**B**). The cells in the control group were treated only with media.

To obtain the polysaccharide fractions, we first performed the decolorization of persimmon leaves using EtOH (Figure 1A). Next, we obtained the crude polysaccharide PLE-0 and performed further fractionation (Figure 1B). Our previous study reported that the elution profile of PLE-0 separated it into three fractions based on the neutral sugar and uronic acid contents [22]. In this study, we estimated the molecular weights of the PLE-0, -I, -II, and -III fractions using high-performance size-exclusion chromatography (HPSEC). HPSEC analysis revealed that the molecular weights of PLE-I–III were 65 kDa (PLE-I), 21 kDa and 13 kDa (PLE-II), and 8 kDa (PLE-III) (Figure 2). In addition, the molecular weight of PLE-0 was in the range of 8–65 kDa, which means that it comprises PLE-I, -II, and -III (Figure S1). Shin et al. previously reported that PLE-0 consists of 71.3% neutral sugars, 26.2% uronic acid, 1.8% KDO-like materials, including 2-O-methyl-fucose, 2-O-methyl-xylose, apiose, aceric acid, 3-deoxy-D-manno-2-octulosonic acid (KDO), and 3-deoxy-D-lyxo-2-heptulosaric acid (DHA), and 0.7% protein [23]. In a subsequent study, Shin et al. purified PLE-0 to PLE-I, -II, and -III and analyzed the monosaccharide composition [22]. In brief, PLE-I mainly consisted of glactose (29.9%), arabinose (17.8%), galacturonic acid (16.7%), rhamose (10.4%), and trace amounts of KDO-like materials (0.9%). PLE-II consisted of 27.2% acidic sugars (galacturnonic acid and glucuronic acid), 19.4% rhamnose, 19.6% arabinose, 13.6% galactose, and 9.6% KDO-like materials such as 2-methyl-fucose (3.0%), 2-methy xylose (3.3%), DHA (0.2%), and KDO (3.1%), which demonstrated RG-II regions of pectic polysaccharides. PLE-III mainly possessed 31.4% acidic sugars, 15.9% rhamnose, 14.6% arabinose, 12.6% galactose, and 1.7% KDO-like materials. Collectively, PLE-I, -II, and -III are pectic polysaccharides, which comprise different sugar components.

### 3.2. Effects of PLE-I–III on HUVEC Viability

Cancer infiltration, progression, and metastasis occur when several staged processes occur continuously [1,2]. Angiogenesis is a major process in cancer development and plays an important role in cancer growth, invasion, and metastasis [2,3,5]. To investigate angiogenesis, we first evaluated the effects of the PLEs (-I, -II, and -III) on the viability of HUVECs using two types of viability assays, the MTT assay system and lactate dehydrogenase (LDH) assay system. As shown in Figure 2, treatment with PLE-I, -II, and -III at concentrations of 6.25 μg/mL to 25 μg/mL for 24 h showed no effect on HUVEC viability. However, treatment with PLE-I and -III at concentrations of 50 μg/mL to 100 μg/mL decreased the viability of HUVECs in a concentration-dependent manner. In addition, treatment with 100 μg/mL PLE-II decreased HUVEC viability. Based on these results, we concluded that treatment with low concentrations (6.25 and 25 μg/mL) of PLEs did not affect the viability of HUVECs, and decided the optimal PLE concentration for the subsequent experiments.

### 3.3. Effects of PLE-I–III on Tube Formation Assay in HUVECs

The tube formation assay performed using vascular endothelial cells such as HUVECs is a representative in vitro angiogenesis evaluation model because HUVECs are capable of capillary-like structures. This process is thought to mimic the process by which endothelial cells form capillaries in vivo [24,25].

**Figure 3:**
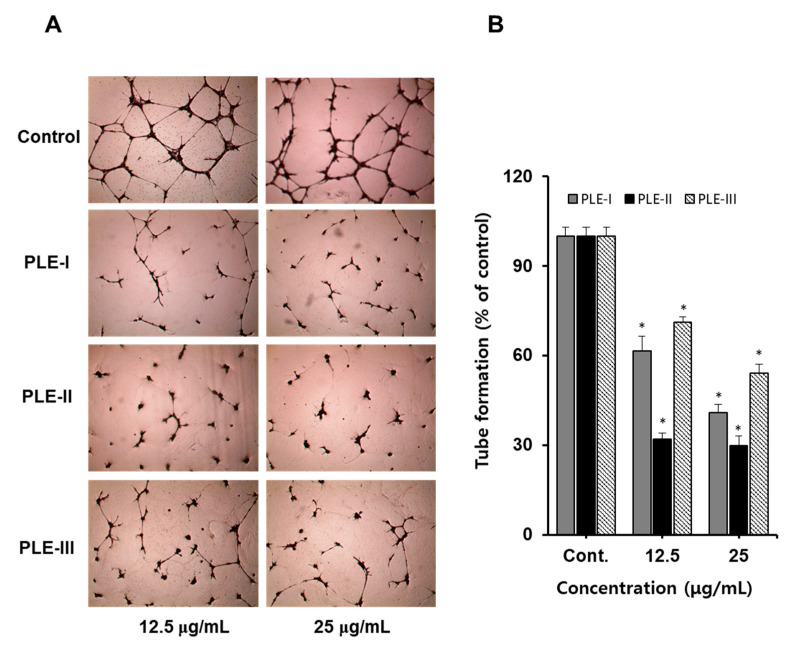
The effect of PLE-I–III on a tubule formation in HUVECs. (**A**) Cells were incubated in PLE-I–III at the indicated concentrations (12.5 or 25 μg/mL) for 24 h. Representative images were obtained using light microscopy after staining HUVECs with hematoxylin. (**B**) The relative length of the tubes was measured using ImageJ software. * *p* < 0.05 vs. the control group.

Therefore, we confirmed the anti-tube formation activity of PLE-I, -II, and -III using HUVECs. As shown in Figure 3A (photographs), treatment with PLE-I, -II, and -III inhibited tube formation in a concentration-dependent manner. Among them, PLE-II strongly inhibited tube formation at 12.5 μg/mL and 25 μg/mL, and PLE-I and PLE-III also showed inhibitory activity. As seen in the quantified bar graph, the inhibition rates of PLE-I, -II, and -III for tube formation were 65.1%, 70.2%, and 45.4%, respectively, at a concentration of 25 μg/mL (Figure 3B). From these data, we predicted that the anti-tube formation property of PLE-II originated from different sugar compositions, including KDO-like materials.

### 3.4. Inhibition of VEGF and MMP-9 Expression in HUVECs by PLE-II

Angiogenesis is regulated by various factors, a representative inducer of which is VEGF. VEGF binds to its receptors (VEGFR-1, -2, and -3) to form dimers and activates the downstream molecules such as Akt, ERK, and p38, ultimately regulating proliferation, migration, and survival [5]. MMPs are a family of enzymes that play important roles in the degradation of extracellular matrix (ECM) components. The ECM lysis process is the first step in cancer invasion and metastasis [10].

**Figure 4:**
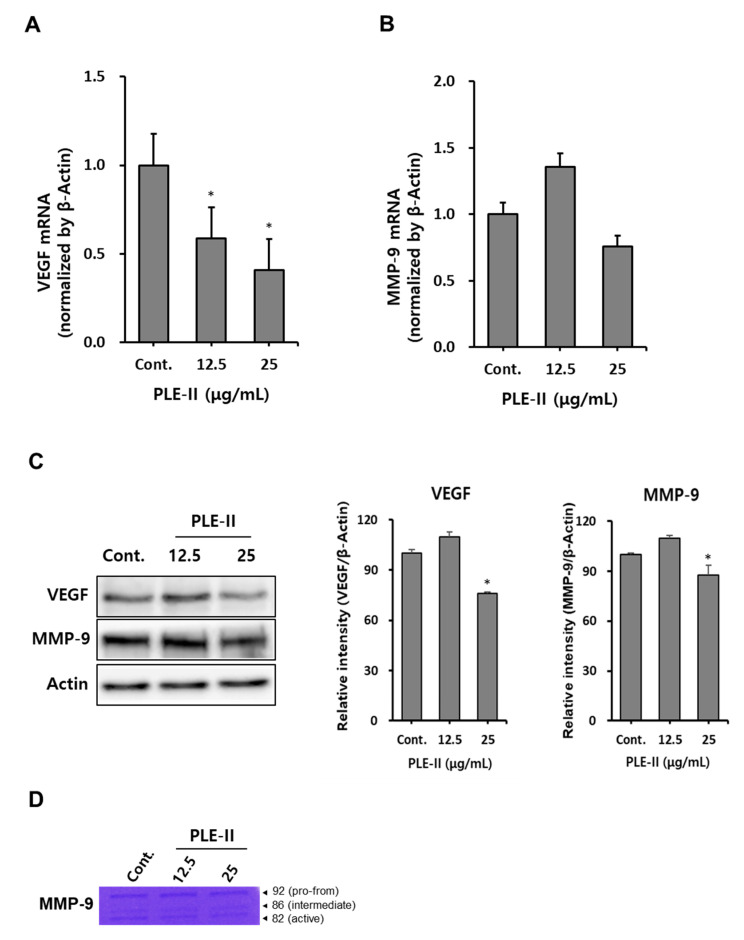
PLE-II polysaccharides suppressed VEGF and MMP-9 expression in HUVECs. (**A**,**B**) HUVECs were seeded at a density of 2.5 × 10^5^ cells/well in a 6-well plate and incubated overnight. Next, the cells were treated with PLE-II at the indicated concentrations (12.5 or 25 µg/mL) for 24 h, and VEGF and MMP-9 mRNA levels were measured using qRT-PCR. (**C**) HUVECs were seeded at a density of 2.5 × 10^5^ cells/well in a 6-well plate then treated with PLE-II at the indicated concentrations for 24 h. Whole cell lysates were then immunoblotted with specific antibodies as indicated in the left side of each panel. β-Actin served as the internal loading control. The bar charts display the intensity of each band after normalization to the intensities of the β-actin bands using the ImageJ software. (**D**) The supernatant was harvested and used for gelatin zymography. * *p* < 0.01 vs. the control group.

Therefore, we evaluated VEGF and MMP-9 mRNA expression after the treatment of HUVECs with PLE-II. As shown in Figure 4A,B, treatment with PLE-II significantly suppressed VEGF mRNA expression in a concentration-dependent manner, and treatment with a high concentration of PLE-II slightly inhibited MMP-9 mRNA expression in HUVECs. Next, we analyzed the protein expression of VEGF and MMP-9 in HUVECs after treatment with PLE-II. Treatment with 25 μg/mL PLE-II significantly suppressed the protein expression of both VEGF and MMP-9 in HUVECs (Figure 4C). Treatment with 25 μg/mL PLE-II inhibited VEGF and MMP-9 expression (inhibition rates of 35.5% and 12.5%, respectively). We also performed zymography to evaluate the activity of MMP-9 in HUVECs after treatment with PLE-II. As shown in Figure 4D, treatment with 25 μg/mL of PLE-II suppressed the conversion of pro-MMP-9 (92 kDa) into active MMP-9 (82 kDa). In addition, we investigated whether PLE-II can downregulate VEGF and MMP-9 expression after treatment with TNF-α. TNF-α is one of the well-known components produced by macrophages, fibroblasts, and endothelial cells in the tumor microenvironment [26,27]. PLE-II treatment strongly downregulated TNF-α-induced VEGF and MMP-9 mRNA expression in HUVECs (Figure S2). Collectively, these results suggest that PLE-II exerts antiangiogenic and antitumor invasion abilities by inhibiting VEGF and MMP-9 expression in HUVECs.

### 3.5. Downregulation of PI3K/AKT, p38, JNK, and NF-κB in HUVECs by PLE-II

**Figure 5:**
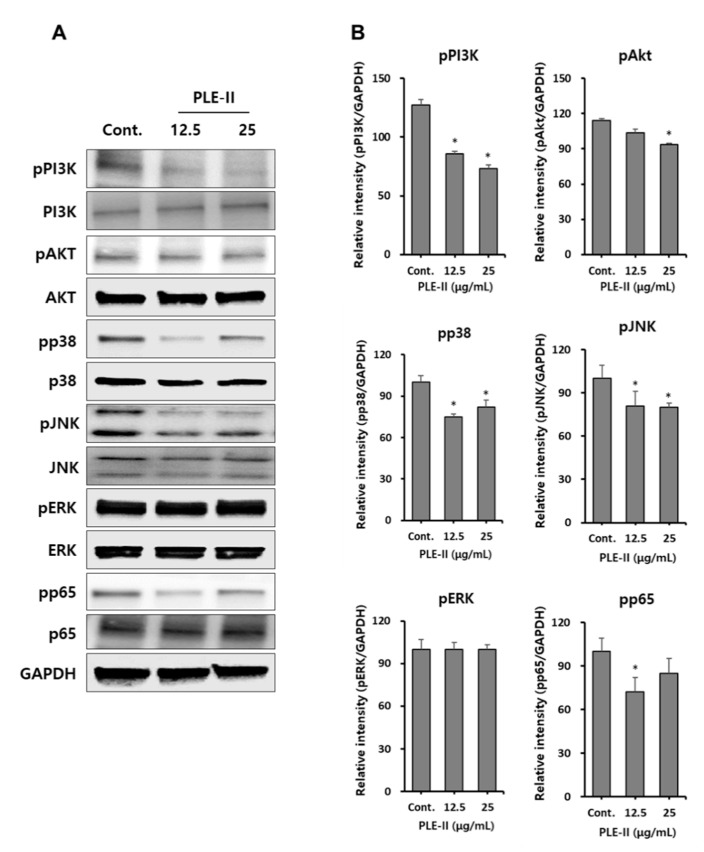
PLE-II inhibited PI3K/AKT, p38, JNK and NF-κB p65 signal pathways in HUVECs. (**A**) Cells were treated with PLE-II at the indicated concentrations for 24 h. Whole-cell lysates were then immunoblotted with specific antibodies as indicated in the left side of each panel. β-Actin served as the internal loading control. (**B**) The bar charts display the intensity of each band after normalization to the intensities of the β-actin using the ImageJ software. * *p* < 0.01 vs. the control group.

VEGFRs are tyrosine kinases that dimerize and can signal through mitogen-activated protein kinases (MAPKs) and AKT. Thus, we attempted to identify the signaling pathways for the suppression of MMP-9 and VEGF by PLE-II in HUVECs. Therefore, we analyzed the signaling pathway molecules such as the PI3K/AKT, MAPKs, and NF-κB signaling pathways. As shown in Figure 5A, treatment with PLE-II inhibited the phosphorylation of PI3K, AKT, p38, JNK, and NF-κB subunit p65 in a concentration-dependent manner. However, the phosphorylation of ERK was not affected by PLE-II treatment (Figure 5A). We also examined the phosphorylation of VEGFR at Tyr-1175 and Tyr-1059, and the total VEGFR expression; however, no significant difference was observed in these parameters following PLE-II treatment for 24 h (data not shown). In addition, we constructed bar graphs of the intensities of the Western blotting bands of the target proteins, following their normalization to the intensities of the β-actin bands (Figure 5B). Collectively, these results suggest that PLE-II exerts antiangiogenic and antitumor invasion abilities mediated by the PI3K/AKT, p38, JNK, and p65 signaling pathways in HUVECs.

## 4. Conclusions

The causes of death in cancer patients are cancer cell metastasis and infiltration rather than the initial tumor. Therefore, it is very important to control cancer cell metastasis. Angiogenesis is an essential process for metastasizing cancer to receive oxygen and nutrients. MMP expression is also considered an important factor in anti-metastatic strategies. Therefore, it is necessary to develop anticancer therapies to inhibit the proliferation of solid tumors by suppressing angiogenesis.

Recently, studies on natural substance-derived polymer substances such as polysaccharides have emerged because polysaccharides are relatively nontoxic. Plant-derived polysaccharides have shown to possess various physiological functions, including anti-metastatic, anti-allergic, and immune-enhancing effects, and are useful candidates for therapeutic development. However, there are many limitations to their development, such as the complexity of the primary structure, monosaccharide composition, and glycosidic linkage analysis, affecting their biological activities. In addition, it is necessary to study the underlying molecular mechanism using specific inhibitors or siRNAs in vitro.

In this study, we showed that the purified pectic polysaccharide PLE-II has antiangiogenic effects mediated by inhibition of VEGF and MMP-9 expression in HUVECs. Moreover, we revealed that the PI3K/AKT, p38, JNK, and NF-κB p65 signaling pathways might contribute to the inhibition of MMP-9 by PLE-II. Taken together, our results suggest that PLE-II, a pectic polysaccharide isolated from the leaves of *Diospyros kaki*, plays a critical role in tube formation which suppresses VEGF, MMP-9 expression in HUVECs. Our data also suggest that PLE-II could be a useful candidate for the development of anti-metastatic agents in the future.

## Figures and Tables

**Table 1:** Primer sequences used for quantitative RT-PCR (qRT-PCR).

Gene Name	Forward Primer	Reverse Primer
VEGF	5′-GCCTTGCCTTGCTGCTCTAC-3′	5′-TTCTGCCCTCCTCCTTCTGC-3′
MMP-9	5′-CGGAGTGAGTTGAACCAG-3′	5′-GTCCCAGTGGGGATTTAC-3′
β-Actin	5′-CCACACTGTGCCCATCTACG-3′	5′-AGGATCTTCATGAGGTAGTCAGTCAG-3′

## Data Availability

Data available in a publicly accessible repository.

## Supplementary Materials

The following are available online at https://www.mdpi.com/2073-4360/13/1/64/s1, Figure S1: Molecular weights of the PLE-0, -I, -II, and -III polysaccharide fractions estimated using a high-performance size-exclusion chromatography (HPSEC) system equipped with Asahipak GS series GS520+GS320+GS220 linked columns. Figure S2: PLE-II inhibits TNF-α-induced VEGF and MMP-9 expression.
