# Supplementary material for: Inhibitory Effects of Pectic Polysaccharide Isolated from Diospyros kaki Leaves on Tumor Cell Angiogenesis via VEGF and MMP-9 Regulation
Source: Polymers (Basel). 2020 Dec 26;13(1):64. doi: 10.3390/polym13010064 (PMC7795685; doi:10.3390/polym13010064)
Supplement: Supplementary file 1 [file polymers-13-00064-s001.pptx]

## Slide 1
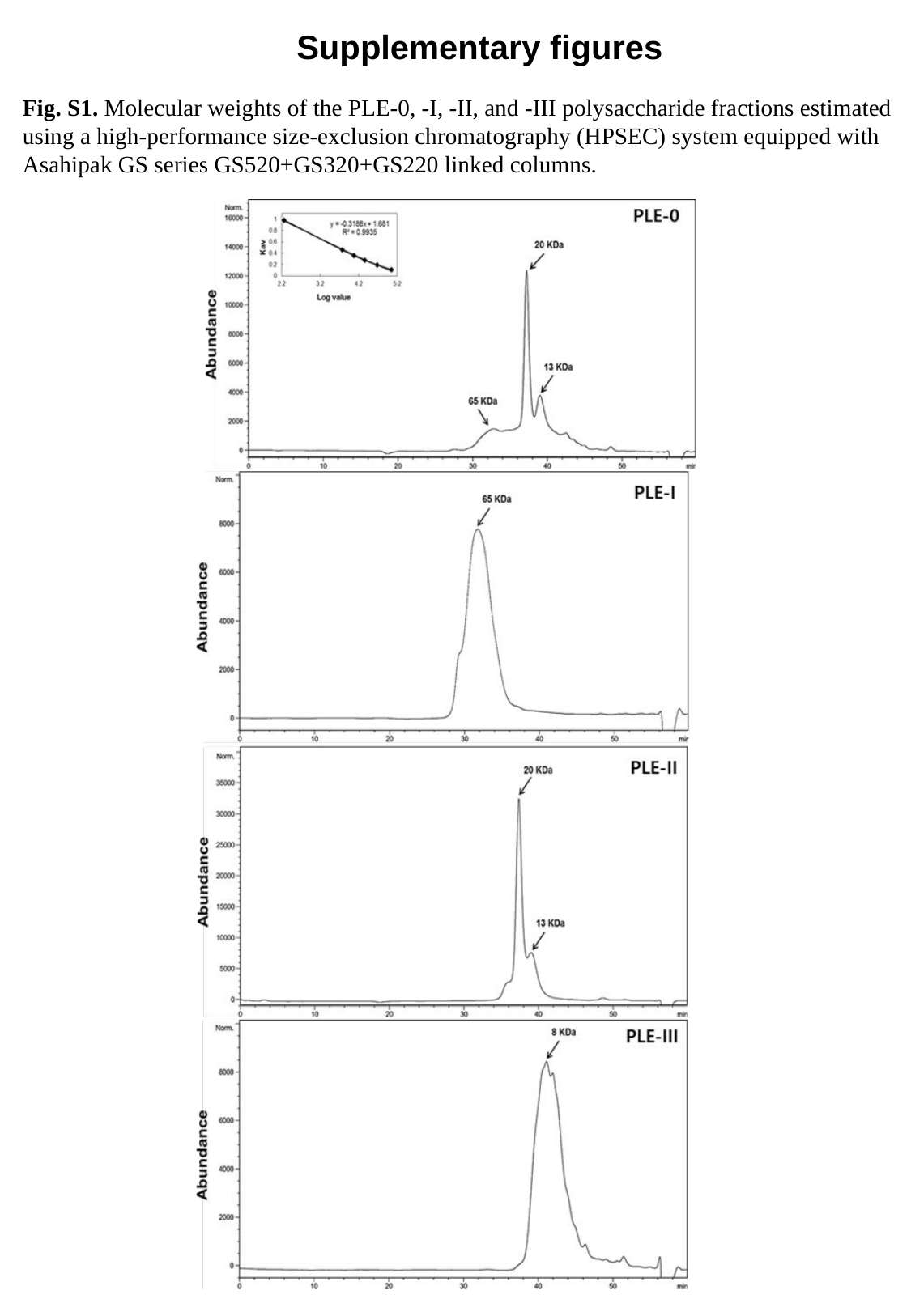

Supplementary figures
Fig. S1. Molecular weights of the PLE-0, -I, -II, and -III polysaccharide fractions estimated using a high-performance size-exclusion chromatography (HPSEC) system equipped with Asahipak GS series GS520+GS320+GS220 linked columns.

## Slide 2
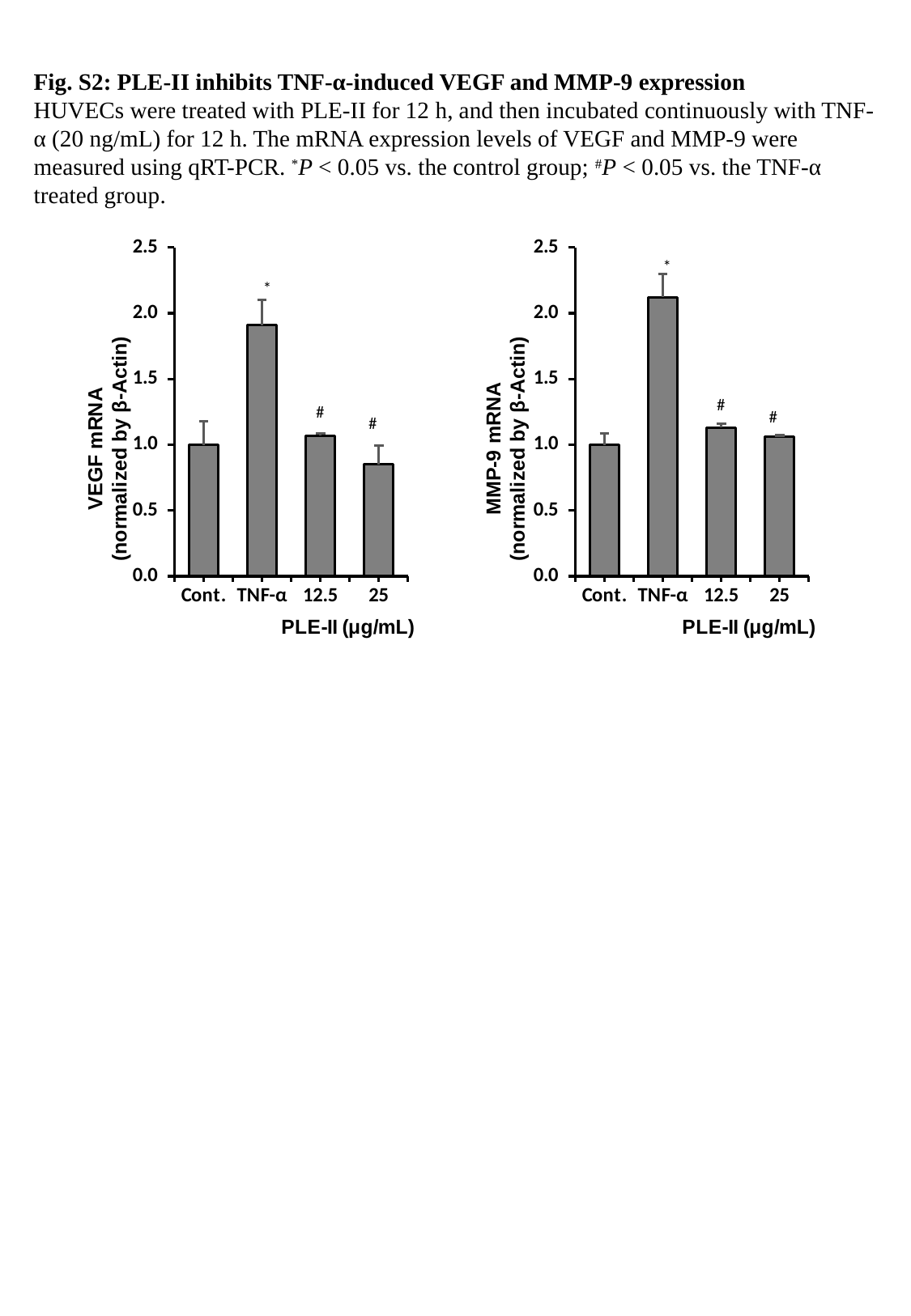

Fig. S2: PLE-II inhibits TNF-α-induced VEGF and MMP-9 expression
HUVECs were treated with PLE-II for 12 h, and then incubated continuously with TNF-α (20 ng/mL) for 12 h. The mRNA expression levels of VEGF and MMP-9 were measured using qRT-PCR. *P < 0.05 vs. the control group; #P < 0.05 vs. the TNF-α treated group.
### Chart
| Category | |
|---|---|
| Cont. | 1.0 |
| TNF-α | 2.12 |
| 12.5 | 1.13 |
| 25 | 1.06 |MMP-9 mRNA
(normalized by β-Actin)
*
#
#
### Chart
| Category | |
|---|---|
| Cont. | 1.0 |
| TNF-α | 1.91 |
| 12.5 | 1.07 |
| 25 | 0.85 |VEGF mRNA
(normalized by β-Actin)
*
#
#
